# An inverse association between tumour size and overdiagnosis may explain the results by Bucchi *et al*

**DOI:** 10.1038/sj.bjc.6602581

**Published:** 2005-04-26

**Authors:** P-H Zahl

**Affiliations:** 1 Norwegian Institute of Public Health, PO Box 4404 Nydalen, Oslo N-0403, Norway

**Sir**,

In a recent paper, Bucchi *et al* (2005) report that the risk of axillary lymph node metastases for screening detected cancers is increasing more rapidly than for clinically detected cancers even after adjusting for other risk factors. They argue that overdiagnosis may not explain their results. I believe that overdiagnosis actually may explain their results.

Further, Bucchi *et al* claim there is ‘no published data support on an inverse association between overdiagnosis and tumour size’. Actually, there are a lot of recent papers on the association between mammography screening and overdiagnosis of small tumours (Olsen and Gotzsche, 2001); (Zahl *et al*, 2004). Most recently, Joensuu *et al* (2004) reported an extremely high level of length time bias in screening detected cancers in the Finnish mammography screening programme.

## References

[bib1] Bucchi L, Barchielli A, Ravaioli A, Federico M, De Lisi V, Ferretti S, Paci E, Vettorazzi M, Patriarca S, Frigerio A, Buiatti E, the SCREENREG Working Group (2005) Screening-detected *vs* clinical breast cancer: the advantage in the relative risk of lymph node metastases decreases with increasing tumour size. Br J Cancer 92: 156–161 15597100 10.1038/sj.bjc.6602289 PMC2361732

[bib2] Joensuu H, Lehtimäki T, Holli K, Elomaa L, Turpeenniemi-Hujanen T, Kataja V, Anttila A, Lundin M, Isola J, Lundin J (2004) Risk for distant recurrence of breast cancer detected by mammography screening or other methods. JAMA 292: 1064–1073 15339900 10.1001/jama.292.9.1064

[bib3] Olsen O, Gotzsche PC (2001) Cochrane review on screening for breast cancer with mammography. Lancet 358: 1340–1342 11684218 10.1016/S0140-6736(01)06449-2

[bib4] Zahl P-H, Strand BH, Maehlen J (2004) Breast cancer incidence in Norway and Sweden during introduction of nation-wide screening: prospective cohort study. Br Med J 328: 921–924 15013948 10.1136/bmj.38044.666157.63 PMC390204

